# KChIP4a is a Biophysical Amplifier of Inhibition in Atypical Dopamine Neurons and Controls Learning from Negative Prediction Errors

**DOI:** 10.1523/JNEUROSCI.1956-25.2026

**Published:** 2026-04-21

**Authors:** Kauê M. Costa, Niklas Hammer-Bahador, Christopher Knowlton, Jochen Schwenk, Tamara Müller, Dorothea Schulte, Bernd Fakler, Carmen C. Canavier, Jochen Roeper

**Affiliations:** ^1^Institute of Neurophysiology, Goethe-University Frankfurt, Frankfurt am Main 60590, Germany; ^2^Department of Psychology, University of Alabama at Birmingham, Birmingham, Alabama, 35233; ^3^Department of Cell Biology and Anatomy, Louisiana State University Health Sciences Center, New Orleans, Louisiana 70112; ^4^Institute of Physiology, University of Freiburg, Freiburg 79104, Germany; ^5^Edinger Institute, Goethe-University Frankfurt, Frankfurt am Main 60590, Germany

**Keywords:** A-type current, dopamine, KChIP4a, Kv4, learning, mesolimbic

## Abstract

Dopamine neurons projecting to distinct brain regions have unique biophysical properties, which are thought to reflect functional specialization. One of these defining properties is the duration of hyperpolarization-induced firing pauses, or rebound delays, which are longer in atypical dopamine neurons targeting ventromedial striatal areas like the nucleus accumbens core. Differences in rebound delay are determined by Kv4 channel-mediated A-type currents, but the mechanisms underlying these differences and their functional implications are not well understood. We hypothesized that KChIP4a, a unique Kv4 β-subunit splice variant, determines rebound delay duration in atypical dopamine neurons. To test this, we generated a transgenic mouse line with dopamine neuron-selective removal of KChIP4a. We found that KChIP4a deletion shortened rebound delays in core-projecting atypical dopamine neurons by changing A-type current kinetics, without affecting conventional dopamine neurons. This indicates that KChIP4a acts as a selective biophysical amplifier of hyperpolarizing inhibition in core-projecting dopamine neurons, and computational modeling suggests that this effect is robust across a wide range of in vivo-like conditions for excitation/inhibition balance. Since firing pauses in core-projecting dopamine neurons are thought to drive learning from reward omission, or negative prediction errors, we tested the effect of KChIP4a deletion on different types of behavior in male and female mice. We found that removal of KChIP4a from dopamine neurons selectively accelerated learning from negative prediction errors, without affecting learning from positive prediction errors or other behavioral variables. Together, our results suggest that KChIP4a fine-tunes subthreshold excitability in projection-defined dopamine neuron populations to regulate specific types of learning.

## Significance Statement

Dopamine neurons that project to different brain areas have distinct cellular properties that are thought to support their specialized roles in behavior. Uncovering the genetic and molecular underpinnings of this functional diversity is critical for understanding how the dopamine system shapes learning and other behaviors in health and disease. We discovered that KChIP4a, a splice variant of a Kv4 potassium channel modulatory subunit, amplifies inhibition in a population of atypical dopamine neurons by changing the kinetics of Kv4 potassium channels. Moreover, removing KChIP4a from dopamine neurons selectively accelerated learning from reward omission, without affecting other behavioral variables. Our findings reveal a novel cell type-specific, alternative splicing-dependent, biophysical mechanism for fine-tuning distinct components of learning.

## Introduction

Midbrain dopamine neurons regulate several behavioral functions, like learning, motivation, and motor control (Costa and Schoenbaum, 2022). They also display a broad range of biophysical properties and gene expression profiles (Poulin et al., 2020; Azcorra et al., 2023), which vary according to their projection target and determine their vulnerability to disease (Farassat et al., 2019; Poulin et al., 2020; Kovacheva et al., 2025; Stojanovic et al., 2025). It has been proposed that the biophysical properties of each subgroup are specialized to support particular behavioral functions (Roeper, 2013). If this is true, then there should also be specific genetic mechanisms underlying such specialization.

Dopamine neurons in the ventral tegmental area (VTA) that project to the nucleus accumbens core (DA-cNAcc), among other “atypical” dopamine neurons (Knowlton et al., 2021), display longer rebound delays after hyperpolarization compared with “conventional” dopamine neurons, like those projecting to the lateral shell of the nucleus accumbens (DA-lNacc) and the dorsolateral striatum (DA-DLS; Lammel et al., 2008). This is one of multiple different properties exhibited by atypical neurons, which also includes smaller afterhyperpolarizations, lower sag currents, higher depolarization block thresholds, and less rebound bursting (Lammel et al., 2008; Tarfa et al., 2017; Knowlton et al., 2021; Stojanovic et al., 2025). Previous work has indicated that slowly inactivating A-type currents mediated by Kv4 channels are in part responsible for the longer postinhibitory delays in atypical neurons (Liss et al., 2001; Tarfa et al., 2017). In conventional dopamine neurons, A-type currents are fast-inactivating and have a different physiological role, which is controlling pacemaker firing rate and stability (Liss et al., 2001; Khaliq and Bean, 2008; Higgs et al., 2023). This suggests that Kv4 channels in atypical dopamine neurons may have a different molecular composition compared with conventional neurons.

Kv4 channels are complexes formed by four pore-forming subunits and whose expression and gating are tightly controlled by auxiliary or β-subunits, including K^+^ channel interacting proteins (KChIPs; Jerng et al., 2004; Jerng and Pfaffinger, 2014; Kise et al., 2021). In mammals, four genes (KCNIP 1–4) encode distinct KChIP proteins, each with multiple splice variants. One of those, KChIP4a, has a unique K^+^ channel inhibitory domain (KID) which slows Kv4 channel inactivation (Holmqvist et al., 2002; Jerng and Pfaffinger, 2014). Most KChIP isoforms, including all other KChIP4 variants, have the opposite effect. The net impact on channel kinetics depends on the relative ratio between KChIP4a and other KChIP isoforms within the channel complex, as each KChIP subunit can bind to one of the pore-forming subunits (Kitazawa et al., 2014; Zhou et al., 2015). Because of these known effects of KChIP4a on Kv4 channels, we hypothesized that this splice variant is the causal determinant of the longer rebound delays seen in atypical dopamine neurons.

Dopamine neuron activity, especially in DA-cNAcc cells, is thought to drive learning by encoding reward prediction errors (RPEs) or the difference between expected and experienced value (Schultz et al., 1997; Costa et al., 2025). This theory posits that pauses in dopamine neuron firing signal negative RPEs, such as omissions of expected rewards. It also predicts that the longer the pauses, the faster the learning from disappointing events, as has been found in previous studies (Bayer et al., 2007; Tian and Uchida, 2015). However, recent findings have challenged this proposition, suggesting that shorter dopamine neuron firing pauses are better drivers of negative RPE-based learning (Chang et al., 2016, 2018; Burwell et al., 2024). While there is controversy on this topic, there is some agreement that reducing firing pauses in dopamine neurons should primarily affect learning based on negative RPEs. Therefore, if our initial hypothesis about the role of KChIP4a is correct, then removing it from dopamine neurons should have a selective effect on learning from negative RPEs.

We tested these hypotheses using a combination of molecular, electrophysiological, behavioral, and computational approaches and demonstrate that KChIP4a expression shapes the longer rebound delays seen in atypical dopamine neurons and learning from negative RPEs.

## Materials and Methods

### Animal protocols

All animal procedures described in this study were approved by the German Regierungspräsidium Gießen and Darmstadt (license numbers: V54-19c 20/15–FU/1100 and FU1203). Mice were bred and housed until 8 weeks of age at MFD Diagnostics. Mice were then maintained under a 12 h dark/light cycle and housed in groups of two to four, with food (R/M-keeping, Ssniff) and tap water available *ad libitum*, except when they underwent water restriction. Nesting material and a red acrylic glass shelter (mouse house, Tecniplast) were used as enrichment. Both males and females were used for all experiments—we did not observe any evident effect of sex in any of the experiments and therefore pooled together data from both sexes. For every single experimental procedure in this study, the experimenter was blind to the genotype of the mice and the order in which mice were tested was pseudorandomized by another team member that was not directly involved in the execution of the experiment.

### The dopamine neuron-specific KChIP4a exon 3 deletion (Ex3d) mouse line

The mouse KChIP4 gene, KCNIP4, is located on chromosome 5 (B3, 48.39–49.52 Mb), is 1,135 kb in length, and has 14 exons, with ATG translation initiation codons in exons 1, 2, 3, 5, and 8, and the STOP codon located in exon 14 (National Library of Medicine; National Center for Biotechnology Information, 2017). KChIP4a (aka KChIP4.4) is a 229 amino acid protein that corresponds to the alternative splicing of exons 3 through 8b of KChIP4, with exon 3 coding for the KID (Holmqvist et al., 2002; Deng et al., 2005).

A new transgenic mouse line was developed for assessing the function of the KChIP4a KID using the Cre-lox system. Exon 3 of the KCNIP4 gene (which encodes the KID) was flanked with loxP sites using a vector designed to display two homology regions in a C57BL/6N genetic background, including a short homology region of 1,795 bp and a long region of 5,858 bp. This vector was electroporated into C57BL/6N embryonic stem cells, and screened clones were used for the generation of mice with homozygotic floxing of KCNIP4 exon 3 (*KChIP4-Ex3^lox/lox^*). These mice were then crossed with a line with heterozygous DAT-Cre knock-in (*DAT-Cre^+/−^* or *DAT-Cre KI*) mice. The offspring of this crossing were then interbred, leading to the production of mice where exon 3 of the KCNIP4 gene was selectively excised from DAT-expressing neurons (Ex3d; *DAT-Cre^+/−^* / *KChIP4-Ex3^flox/flox^*) and DAT-Cre KI control littermates (CTRL; *DAT-Cre^+/−^ / KChIP4-Ex^WT/WT^*).

Genetic identity of all KChIP4-Ex3^flox/flox^ mice was confirmed with polymerase chain reaction (PCR) genotyping throughout the breeding process. For detecting the conditional deletion allele, the following primers were used:

5-TAG TTA TGA CAA GAC AGG AGC TAG TAC CAC TAA GC-3

and

5-GAA CTG GAC TGA AGC AAA ACA AAA CAC G-3.

These primers target the flanking region of the FRT site, and the PCR product lengths were 385 bp for the WT allele and 477 bp for the conditional deletion allele. The PCR protocols were run with 1 cycle at 94°C for 120 s (denaturing), 35 cycles of 94°C for 30 s (denaturing), 65°C for 30 s (annealing) and 68°C for 30 s (extension), followed by 1 cycle at 68°C for 480 s (completion).

### FISH followed by immunofluorescence detection

All steps were conducted at room temperature unless otherwise indicated. Fourteen micrometer-thick coronal cryosections were postfixed for 5 min in 4% PFA/PBS, washed three times in PBS [0.1%, diethyl pyrocarbonate (DEPC)] for 5 min, and washed once in 1× saline-sodium citrate (SSC, 0.1% DEPC) for 5 min. Prehybridization was performed with 200 µl of hybridization buffer (50% formamide, 5× Denhardt’s solution, 5× SSC, 0.25 mg/ml yeast tRNA, 0.2 mg/ml salmon sperm DNA, DEPC-H2O) per slide for 4 h. Hybridization was performed with 1 ng/µl digoxigenin (DIG)-labelled probes overnight at 65°C. Probes corresponded to (1) KCHIP4a: NT 1-192 of *Mus musculus* KCNIP4, transcript variant 4 (NCBI NM_030265.3) and (2) KCHIP4 all: NT 455-839 of *Mus musculus* KCNIP4, transcript variant 4 (NM_001199242.1). DIG-labeled antisense probes were generated following standard procedures. Posthybridization washes were performed at 60°C for 5 min in 5× SSC, followed by 5 min in 2× SSC, and 20 min in 0.2× SSC/50% formamide. Slides were allowed to cool down for 20 min and washed twice in 0.2× SSC. Endogenous peroxidase activity was quenched by incubation in 3% H_2_O_2_/1× SSC for 15 min. Slides were washed twice for 5 min in 1× TBS followed by 30 min blocking in TNB blocking buffer [0.1 M Tris-HCl, 0.15 M NaCl, 0.5% (w/v) TSA blocking reagent (PerkinElmer)]. Slides were incubated, in a humidified chamber,in anti-DIG-POD Fab fragments (1:500; Roche) diluted in TNB buffer for 2 h, followed by washing three times in TBS-T (0.1% Tween-20) for 10 min. Tyramide signal amplification was performed with 100 µl of TSA Plus FITC (1:60; PerkinElmer) per slide for 10 min in a dark chamber. Sections were incubated with Ar6 buffer (1:10; PerkinElmer) for 45 min in a heat steamer and allowed to cool down for 30 min.

Immunohistochemistry was performed following standard procedures. Briefly, slides were blocked for 30 min in 0.3% Triton/10% normal goat serum/TBS followed by overnight primary antibody incubation (1:1,000; rb TH; Merck Millipore) at 4°C. Slides were washed three times for 10 min in TBS-T, and secondary antibody incubation (1:750; Life Technologies) was performed for 1 h. After washing three times in TBS-T for 10 min, slides were incubated in 1× DAPI for 10 min, washed three times for 5 min with TBS, and embedded in ProLong Gold Antifade Mountant (Thermo Fisher Scientific). Images were taken with a Nikon Eclipse90i microscope, and acquisition was performed with the NIS-Elements software (version 5.01). Brightness and contrast were moderately enhanced in Adobe Photoshop across the entire image, and no further image processing was performed.

### Kv4 channel complex high-resolution proteomics

#### Crude membrane preparation and protein solubilization

Isolated cerebellum and midbrain samples of six CTRL and six Ex3d mice were individually processed. Tissues were homogenized two times in 0.5 ml of homogenization buffer (in mM: 320 sucrose, 10 Tris/HCl, 1,5 MgCl2, 1 EGTA, 1 iodoacetamide, pH 7.5) supplemented with protease inhibitors [aprotinin, pepstatin, leupeptin (each at 2 µg/ml) and 1 mM PMSF] with a Dounce homogenizer. Homogenates were centrifuged at 100 × *g* for 5 min and resulting supernatants centrifuged at 200,000 × *g* (S45A rotor, Sorvall) for 20 min. Pellets were resuspended in 0.5 ml of lysis buffer (in mM: 5 Tris/HCl, 1 EDTA, 1 iodoacetamide, protease inhibitors) and after 30 min incubation time on ice centrifuged at 200,000 × *g* for 20 min. The resulting pellets were resuspended in 0.1 ml of 20 mM Tris/HCl pH 7.5 and protein concentrations determined by Bradford assay. Then, 0.3 mg of each sample was solubilized in 0.5 ml of CL-48 buffer (Logopharm) with freshly added protease inhibitors for 30 min on ice. Nonsolubilized material was removed by ultracentrifugation (8 min at 125,000 × *g*, S45A).

#### Affinity purifications

Solubilizates were directly incubated with 7.5 µg Kv4.3 antibodies (Alomone Labs, #APC-017) immobilized on Protein A Dynabeads (Invitrogen) for 2 h at 4°C. Two additional 0.5 ml of solubilizates of cerebellum and midbrain samples were incubated with 7.5 µg IgG (Millipore, 12-370), which were used as internal specificity control. After two washing steps with 0.5 ml of CL-48 buffer, bound proteins were eluted with 1× Laemmli buffer without DTT. Proteins were shortly run on 10% SDS-PAGE and silver stained. Samples were further processed as described (Schwenk et al., 2012), and lanes were split into two pieces and digested with sequencing-grade modified trypsin (Promega, #V5111) and alpha-lytic protease (Sigma-Aldrich, #A6362). Peptides were extracted and prepared for MS analysis.

#### Mass spectrometry

Mass spectrometric analyses of peptide mixtures were carried out on a Q Exactive HF-X mass spectrometer coupled to an UltiMate 3000 RSLCnano HPLC system (both Thermo Fisher Scientific) as described previously (Schwenk et al., 2019). For each LC-MS/MS dataset, a peak list was extracted from fragment ion spectra using the “msconvert.exe” tool (part of ProteoWizard; http://proteowizard.sourceforge.net/; v3.0.6906; Mascot generic format with filter options “peakPicking true 1-” and “threshold count 500 most-intense”), and the precursor m/z values were shifted by the median m/z offset of all peptides assigned to proteins in a preliminary database search with 50 ppm peptide mass tolerance. Corrected peak lists were searched with Mascot (Matrix Science) against all mouse, rat, and human entries of the UniProtKB/Swiss-Prot database (supplemented with mouse KChIP4 splice isoforms, identifier: Q6PHZ8-2, Q6PHZ8-3, Q6PHZ8-4, Q6PHZ8-5, Q6PHZ8-6). Acetyl (Protein N-term), carbamidomethyl (C), Gln->pyro-Glu (N-term Q), Glu->pyro-Glu (N-term E), oxidation (M), and propionamide (C) were chosen as variable modifications, and peptide and fragment mass tolerance were set to ±5 ppm and ±0.8 Da, respectively. One missed cleavage was allowed. The expect value cutoff for peptide assignment was set to 0.5.

#### Protein quantification

Label-free quantification of proteins was done as described previously (Bildl et al., 2012; Muller et al., 2016). Peptide signal intensities (peak volumes, PVs) were determined and offline mass calibrated using MaxQuant (http://www.maxquant.org) and then assigned to peptides based on their m/z and elution time obtained either directly from MS/MS-based identification or indirectly (i.e., from identifications in parallel datasets) using in-house developed software. Molecular abundances of proteins were estimated using the abundance_norm, spec_ score defined as the sum of all assigned and protein isoform-specific PVs divided by the number of MS-accessible protein isoform-specific amino acids (Bildl et al., 2012). In order to visualize molecular compositions of Kv4-KChIP complexes, normalized molecular abundance values of Kv4.2, Kv4.3, KChIP1, KChIP2, KChIP3, KChIP4, DDP6, and DPP10 were calculated (Fig. 3*B*,*C*; Fig. S1). Normalized values were obtained by dividing abundance_norm,spec_ values of individual proteins by the factor (∑ of Kv4 abundances/4). Hence, the sum of normalized molecular abundance values for Kv4.2 and Kv4.3 is 4. Relative abundances of KChIP4a isoform in Ex3d versus CTRL samples were determined by use of PV values of 3–5 isoform-unique peptide features. For each unique peptide, the PVs were first normalized to their maximum over 12 AP datasets from cerebellum and over 12 AP datasets from midbrain, yielding relative peptide profiles. Accordingly, relative abundance differences of KChIP4a were determined as mean value of peptide profiles from six Ex3d samples and six CTRL samples from cerebellum and midbrain, respectively, whereby the CTRL was set the value of 1 (Fig. 3*D*).

### Patch-clamp recordings of projection-identified dopamine neurons

#### Retrograde tracing

Mice were premedicated with carprofen (Rimadyl, Pfizer, 5 mg/kg, s.c.). Anesthesia was induced and maintained throughout the surgery using isoflurane (AbbVie; induction, 4% in 350 ml/min O2; maintenance dose, 1–2.5% in 350 ml/min O_2_). For local anesthesia and analgesia, we administered a mixture of lidocaine and prilocaine (EMLA, Aspen). After induction of anesthesia, mice were transferred in a stereotaxic frame (Kopf Instruments) where 100 nl of red retrobeads (diluted 1:30 in aCSF, Lumafluor) were injected in different areas of the striatum (in reference to bregma: nucleus accumbens core: AP: 1.54 mm, ML: ±1.0 mm, DV: 4.0 mm; nucleus accumbens lateral shell: AP: 0.86 mm, ML: ±1.75 mm, DV: 4.5 mm; DLS: AP: 0.74 mm, ML:± 2.2 mm, DV: 2.6 mm) to label midbrain dopamine neurons, as previously described (Lammel et al., 2008).

#### Ex vivo electrophysiology

After 3 d of recovery, to ensure sufficient retrograde axonal transport of the red retrobeads, the mice were perfused transcardially using ice-cold perfusion solution (in mM: 125 NaCl, 2.5 KCl, 6 MgCl_2_, 0.1 CaCl_2_, 25 NaHCO_3_, 1.25 NaH_2_PO_4_, 50 sucrose, 2.5 glucose, 3 kynurenic acid, bubbled with carbogen). Brains were quickly removed, and the midbrain was sliced into 250-µm-thick coronal slices using a vibratome (Leica VT1200S, Leica Biosystems). Slices were then transferred to a beaker containing ACSF (in mM: 125 NaCl, 3.5 KCl, 1.2 MgCl_2_, 1.2 CaCl_2_, 25 NaHCO_3_, 1.25 NaH_2_PO_4_ constantly bubbled with carbogen) and allowed to recover at 37°C for 60 min.

After recovery, slices were transferred to a recording chamber and constantly perfused at a rate of 2–4 ml/min^−1^ with ASCF maintained at 37°C (temperature controller VI, Luigs & Neumann). Synaptic transmission was blocked using CNQX (12.5 μM, Biotrend), dʟ-AP5 (10 μM, Tocris), and gabazine (4 μM, SR95531, Biotrend). Midbrain dopamine neurons were visualized using a light microscope (Axioskop 2 FS plus, Zeiss) and an infrared camera (VX55, TILL Photonics). Red retrobeads were excited by an epifluorescence lamp (HBO100 Nikon) filtered at 546/12 nm. Dopamine neurons containing red retrobeads were recorded using borosilicate pipettes (3–4 MOhm, GC150TF, Harvard Apparatus) filled with internal solution (in mM: 135 K-gluconate, 5 KCl, 10 HEPES, 0.1 EGTA, 5 MgCl_2_, 0.075 CaCl_2,_ 5 ATP, 1 GTP, 0.1% Neurobiotin, pH 7.35, 290–300 mOsmol). Data were recorded at 20 kHz and filtered with a low-pass filter Bessel characteristic of a 5 kHz cutoff frequency using an EPC 10 amplifier (HEKA Elektronik). Patchmaster (HEKA Elektronik), Igor Pro (WaveMetrics), and MATLAB (MathWorks) were used for acquisition and analysis. Only spontaneously active cells that showed robust pacemaking, and that were post hoc identified as TH positive and containing red retrobeads, were included in this study.

#### Histology

The forebrain tissue-block containing striatal injection sites, as well as midbrain slices containing the recorded and neurobiotin-filled neurons, was immersion-fixed at 4°C overnight in a solution of 4% paraformaldehyde and 15% picric acid in phosphate-buffered solution (PBS). Striatal injection sites were cut into 100 µm coronal sections using a vibratome (VT1000S, Leica Biosystems). Free-floating sections were washed in PBS and incubated with blocking solution (10% horse serum, 0.5% Triton X-100, and 0.2% BSA in PBS) at room temperature for 1 h. Afterward, sections were incubated in carrier solution (1% horse serum, 0.5% Triton X-100, and 0.2% BSA in PBS) containing the primary antibody (polyclonal rabbit anti-TH, 1:1,000, Synaptic Systems, catalog #213 104) overnight at room temperature. On the second day, sections were washed several times in PBS and incubated with carrier solution containing the secondary antibody (goat anti-rabbit 488, 1:750, Thermo Fisher Scientific, catalog #A-11011) and in case of the midbrain sections Streptavidin Alexa Fluor 408 (1:750, Invitrogen) overnight at room temperature. On the third day, sections were washed in PBS, mounted, and stored at 4°C. Images were taken with a Nikon Eclipse90i microscope, and acquisition was performed with the NIS-Elements software (version 5.01). The spread of retrobeads in the striatum, as displayed in Figure S2, was extracted by applying a mask on the red channel of the acquired microscope images in FIJI (Schindelin et al., 2012) and then pasting that over a schematic of the striatum. The location of the recorded neurons in the midbrain, as displayed in Figure S2, was estimated visually based on the acquired images and plotted over a midbrain section schematic.

### Biophysical modeling of dopamine neurons

#### Model calibration

The single compartment, core-projecting dopamine neuron model was adapted from Knowlton et al. (2021). The inactivation variable of the Kv4.3 channel model from that study was separated into an additive combination of slow and fast variables. Steady-state activation, inactivation, and kinetics of inactivation were set to be consistent with voltage-clamp data in Figure 5 and are given in Table 1. Activation kinetics was not altered from those originally adapted from Tarfa et al. (2017) and was assumed to be identical for channels with slow or fast inactivation gates. Steady-state activation and inactivation are given by Boltzmann functions, while the inactivation time constants are given by Boltzmann sigmoids between indicated minimum and maximum values. The relative contributions of the slow and fast Kv4.3 channels, determined by the binding of different auxiliary subunits, is the only intrinsic model parameter varied between CTRL and Ex3D models. The model was implemented in NEURON (Hines and Carnevale, 2001; Hines et al., 2009) with extensions for parallel computing (Migliore et al., 2006). Model files are freely available at https://modeldb.science/2019874 (password: okaychip).

**Table 1.** Steady-state activation, inactivation, and kinetics of inactivation of the Kv4 currents of the biophysical models of atypical dopamine neurons

	Activation (*p*^3^)	Inactivation (*q*)	*q*Tau (slow)	*q*Tau (fast)	*f*_s_
*V*_half_ (mV)	−40.8	−68.0	−60	−60	CTRL: 56:44
*V*_slope_ (mV)	7.73	−6.44	5	4	
Min, max	–	–	20, 250 ms	20, 45 ms	Ex3d: 22:78

#### Balanced state methods

A simulated in vivo balanced state was implemented by simultaneous application of high-frequency, low-conductance (100 Hz) Poisson GABA_A_ and NMDA receptor activation (Komendantov et al., 2004; Canavier and Landry, 2006; Canavier et al., 2016; Knowlton and Canavier, 2023). Glutamate activation consists of simultaneous NMDA and AMPA EPSCs, with 0.3 AMPA to 0.7 NMDA peak conductance (Lammel et al., 2011). The NEURON implementation of NMDA receptor activation was taken unmodified from Moradi et al. (2013). AMPA and GABA_A_ receptor activation uses NEURON’s built-in biexponential synapse both with a rise time of 0.2 ms and fall times of 2.4 and 3 ms, respectively. The reversal potential of chloride was fixed at −60 mV for all balanced state GABA_A_ and the reversal potential of NMDA and AMPA at 0 mV. The Ca^2+^ influx via NMDA channel was not included in the pool that activates the SK K+ current. Balanced state synaptic weights for AMPA, NMDA, and GABA_A_ were set to be constant within each trial. These weights were chosen to obtain a simulated in vivo firing rate was consistent with in vivo recordings, and the GABA_A_ conductance was of sufficient magnitude to silence cells in the absence of excitation, consistent with a balanced state. Conductances, but not rates, were varied in concert from 125% glutamate/75% GABA to 75% glutamate/125% GABA to produce a spread of balanced state firing rates covering the full range of observed in vivo firing.

Inhibitory synaptic inputs, as would putatively underlie the signaling of negative RPEs, were modeled as additional GABA_A_ inputs applied synchronously. Inhibition trials were repeated then recombined into a histogram of simulated network response under an ergodic approximation. Each histogram was constructed from 500 simulated trials. An intertrial refractory period of 4 s was used to eliminate transients. Multithreading was used to run different parameterizations of the simulated RPE trials.

### Behavior

#### Open field

Spontaneous locomotor and exploratory activity (track length, wall distance, time in center, and number of rearings) were evaluated in an open field arena (a lidless box measuring 52 × 52 cm, under red illumination at 3 lux) using a video tracking system (Viewer II, Biobserve) as described in a previous study from our group (Schiemann et al., 2012). For a comprehensive phenotyping of mutant mice, the total track length, the time spent and track length within the center area of the arena (defined as a 30 × 30 cm square zone with all sides equidistant from the walls), and the number and duration of rearings were evaluated. Rearings were recorded via infrared beam breaks at a height of 4.5 cm and defined by being at least 200 ms long and two subsequent rearings had to be at least 80 ms apart. After 10 min, the mice were returned to their home cages for at least 2 min and then subjected to the novel object recognition test.

#### Novel object recognition

A novel object exploration and preference task was used to assess the mice's recognition, memory retention, and preference for novel stimuli (Ennaceur and Delacour, 1988; Antunes and Biala, 2012). In this task, mice were placed in the same open field arena described in the previous section, after the open field test (which also served as a habituation for the novel object recognition test), but within the arena two identical objects (stainless steel cylinders; 3 cm diameter × 6 cm height) were placed at equal lengths from each other and at 15 cm from the upper left and right corners. The mice were allowed to freely explore the arena and the objects for 10 min (Trial 1), after which they were removed from the arena. Subsequently, one of the objects was replaced by a different, novel object (plastic coated rectangular prism; 3 × 3 cm base × 6 cm height), and the mice were again allowed to explore the arena and the objects (Trial 2).

Object recognition was analyzed using Biobserve's Object Recognition plug-in, and object interaction events were defined as periods in which a mouse was directly facing the object (snout directed to the object within a 180° angle) at a distance shorter than 3 cm. Both the number and duration of these interaction events were quantified for both trials of the task. Exploration dynamics (number and duration of objected-directed exploration) were analyzed for both trials, as well as the differences between exploration of an already explored (“old”) and novel (“new”) object in Trial 2 were analyzed. For the quantification of novel object discrimination and preference, a discrimination index was calculated by dividing the difference between the time spent exploring the novel object and the time spent exploring the familiar object by the sum of these two measures (Ennaceur and Delacour, 1988; Antunes and Biala, 2012):

$$\mathrm{DI}=\;\frac{T_{\mathrm{New}}-T_{\mathrm{Old}}}{T_{\mathrm{New}}+T_{\mathrm{Old}}},$$

where

DI = discrimination index

*T*_New _= time spent exploring the novel object

*T*_Old_ = time spent exploring the familiar object

#### Hole board

Exploration of holes, a naturalistic behavior for mice, was evaluated with the hole board task (Crawley, 2007). In this task, mice were placed for 5 min in an open field arena similar to the one used for the open field test, but with four circular holes (2 cm in diameter) on its floor (ActiMot2, TSE Systems). When confronted with such an arena, mice tend to spontaneously check the holes by dipping their heads in them. The performance of head dips was recorded with infrared beam breaks placed immediately under the inferior surface of the floorboard. The latency to the first head dip, as well as the total number and duration of head dips, was quantified. The percentage of repeated head dips (two dips performed sequentially into the same hole) as well as the percentage of dips into the preferred hole (the hole that was most explored during the session) were also quantified and compared between genotypes.

#### Spontaneous alternation in the plus maze

Working memory performance was quantified between genotypes using the spontaneous alternation in the plus maze (Ragozzino et al., 1996). Mice were placed in a plastic plus maze (four equidistant 35 × 4.5 cm arms radiating at 90° angles from a circular central arena with 10 cm diameter; walls were 15 cm in height) and allowed free exploration for 12 min (Lennartz, 2008). An arm entry was defined as when the mouse enters an arm with all its four paws. Each session was recorded with a video camera, and quantification of arm entries was made through visual analysis of the videos. A spontaneous alternation was marked when the mouse explored four different arms in five consecutive arm entries, and the proportion of spontaneous alternations was quantified as the number of real alternations divided by the total possible number of alternations (sum of all arm entries minus 4); chance performance in this task is calculated to be 44% (Ragozzino et al., 1996; Lennartz, 2008).

#### Reinforcement learning task

Male and female Ex3d (*N* = 10) and CTRL (*N* = 20) mice, over 8 weeks old, were genotyped and individually identified by the implantation of a subcutaneous transponder microchip (1.4 mm × 9 mm ISO FDX-B glass transponder, Planet ID). Behavioral comparisons were performed between Ex3d and DAT-Cre KI mice. One Ex3d mouse showed erratic behavior within the conditioning box and was excluded from data analysis (final N of Ex3d group = 9). All mice were littermates (hence the difference in number between the CTRL and Ex3d groups), and experiments were replicated across three separate cohorts.

Mice were motivated by water restriction (∼85% of their initial body weight) and were rewarded with a solution of 10% sucrose in tap water. Daily water rations varied between 1 and 1.5 ml depending on the mice’s weight on that day, with a set target of 85% of the initial body weight. Except during experimental sessions, water was always delivered in a cup placed in their home cage. Mice were also closely monitored to ensure that the water supply was consumed and not spilt over or contaminated and the health of the water restricted mice was also evaluated daily (Guo et al., 2014). This protocol maintained a stable body weight around the desired target during all experimental sessions and did not result in signs of overt dehydration or ill health (e.g., hunched posture or ruffled fur).

The full experimental paradigm spanned 24 d. On the first 4 d, mice were submitted only to water restriction. In the following 2 d, they were tamed, i.e., gently handled (held up above their home cage on a spread palm, with no constriction or entrapment by the experimenter) until they no longer tried to escape from the experimenter's hand, showed no overt signs of stress and anxiety, and readily drank a portion of liquid reward (0.2 ml) given by the experimenter via a syringe while being held. The following day, the mice were placed inside the operant chamber with the reward port removed for 50 min in order to acclimate to the experimental conditions. After this time, they were returned to their home cage and given their daily ration of water. The day after that, mice underwent shaping, i.e., they were placed in the operant chamber for another 50 min, now with the reward port present, and at semirandom time intervals (mean of 60 s, varying between 30 and 90 s), a reward portion (16.66 µl) was delivered at the port, with no cue to its delivery. The following day, mice were submitted to the conditioning task.

The task used in this study is based on the study by Steinberg et al. (2013). In this paradigm, mice learned to associate a CS (sound tone pulsed at 3 Hz—0.1 s on/0.2 s off—at 70 dB) to the availability of reward in the reward port. Each session consisted of 10 trials (mean intertrial interval of 4 min, varying between 1.5 and 6.5 min) in which the auditory cue was on for 30 s. Mice could trigger reward delivery to the port by entering it during CS presentation. Rewards were delivered in a cycle of 2 s reward delivery (16.66 µl) followed by a 3 s consumption interval. Delivery was continuous for as long as the mouse kept its head in the port during CS presentation. This allowed for a maximum of 6 rewards per trial and a maximum of 60 rewards per session (a total of ∼1 ml or reward in the task per day). Additional water supplementation, when needed to complete the mice’s daily water ration, was provided in the home cage as described in the previous paragraph.

This acquisition phase lasted for 11 daily sessions. After acquisition, extinction of the conditioned response was tested by consistently omitting the reward during CS presentation for six daily sessions. After extinction, mice were returned to their home cage and received water *ad libitum* for at least 3 d before being submitted to other behavioral tasks. All mice recovered their initial body weight in this period and showed no long-term adverse effects from water restriction.

Performance in this task was quantified by the total time the mice spent in the port during the cued trials and the latency to enter the port after cue onset. Time in port was normalized both as a percentage of total reward availability time and also by subtracting the amount of time the mouse spend in the port 30 s before cue onset (Steinberg et al., 2013). This means that CS–US associations were quantified by the mouse responding both quickly and selectively to the cue. In addition, the time stamps of every head entry and head retraction into the port were recorded, which allowed the quantification of the dynamics of head entries during cue presentation and during the ITI. Latency was quantified as the time between cue initiation and the first head entry into the reward port; if mice did not respond in a trial, the maximal possible latency value (30 s) was ascribed to that trial. The dynamics of head entries during extinction were used to infer how the mouse adapts its behavioral response in the absence of an expected reinforcement, i.e., whether it perform fewer head entries (a proxy of the initiation rate of reward-seeking behaviors), head entries that are just shorter in length (indicating a faster disengagement from reward-seeking behaviors), or a combination of both.

### Behavioral modeling

A modified Rescorla–Wagner model, which applied two different learning rates for positive (*α*_P_) and negative (*α*_N_) RPEs, was used in order to formally quantify differences in learning between Ex3d and CTRL genotype groups (Rescorla and Wagner, 1972; Raczka et al., 2011; Tian and Uchida, 2015). In detail:

$$\mathrm{if}\;\;(R-V_{n})\;\geq 0\;[\mathrm{positive}\;\mathrm{RPE}],$$

$$V_{n\;+\;1}=V_{n}+\alpha_{P}(R-V_{n}),$$

$$\mathrm{if}\;\;(R-V_{n})<0\;[\mathrm{negative}\;\mathrm{RPE}],$$

$$V_{n+1}=V_{n}+\alpha_{N}(R-V_{n}),$$

where

*V* = associative strength between CS and US

*α*_P_ and *α*_N_ = learning rates from positive and negative RPEs, respectively

*R* = maximal associative strength of the US (reward value)

*n* = trial index

While the Rescorla–Wagner model cannot recapitulate long-term features of extinction learning, like spontaneous recovery and renewal (Redish et al., 2007; Pan et al., 2008), it is sufficient to reconstruct the immediate, short-term inhibitory behavioral effects of extinction (Raczka et al., 2011). Moreover, while the Rescorla–Wagner model assumes that learning from negative prediction errors is a form of unlearning, we do not make the same assumption; we only use the model to quantify learning rates from positive and negative RPEs using a simple prediction error rule. All modeling and fitting were done with custom MATLAB code. The percentage of time in port metric was assumed to be a linear readout of *V*. The limits of *V* in relation to the empirical data were bound by the initial value of *V* before the task (*V*_0_), the lower and upper limits of associative strength readout (*V*_min_ and *V*_max_, respectively). These parameters were derived empirically from each mouse, with *V*_0_ being set as the average response in the first three trials of acquisition, and *V*_min_ and *V*_max_ being set as the average responses in the last two sessions of acquisition and extinction, respectively. Fitting of learning rates was performed with least absolute residuals method by implementing the *fminsearch* MATLAB function (Tian and Uchida, 2015). Goodness of fit was inferred from the residual values of the optimal fit for each mouse.

### Statistical analyses

For two-group comparisons, data were tested for normality with the Kolmogorov–Smirnov test. If both distributions were Gaussian, differences were analyzed using two-tailed *t* tests; otherwise, the two-tailed Mann–Whitney test was used. One-tailed tests were used when there was a specific directional hypothesis. Comparisons between more than two groups were tested using one-way ANOVA. Multivariate comparisons were evaluated using two-way repeated-measures ANOVA with Sidak's multiple-comparisons post hoc tests. Cells recorded from different mice were all analyzed as individual samples, but we also replicated the analyses using nested *t* tests with individual mice as a nested factor to ensure genotype differences were not due to individual variance within groups. Statistical significance was set at *p* < 0.05 for all comparisons. Unless otherwise noticed, data is expressed as mean ± SEM. The figures were created with MATLAB, GraphPad Prism, and BioRender.

## Results

### Deletion of KChIP4a from midbrain dopamine neurons

**Figure 1.**
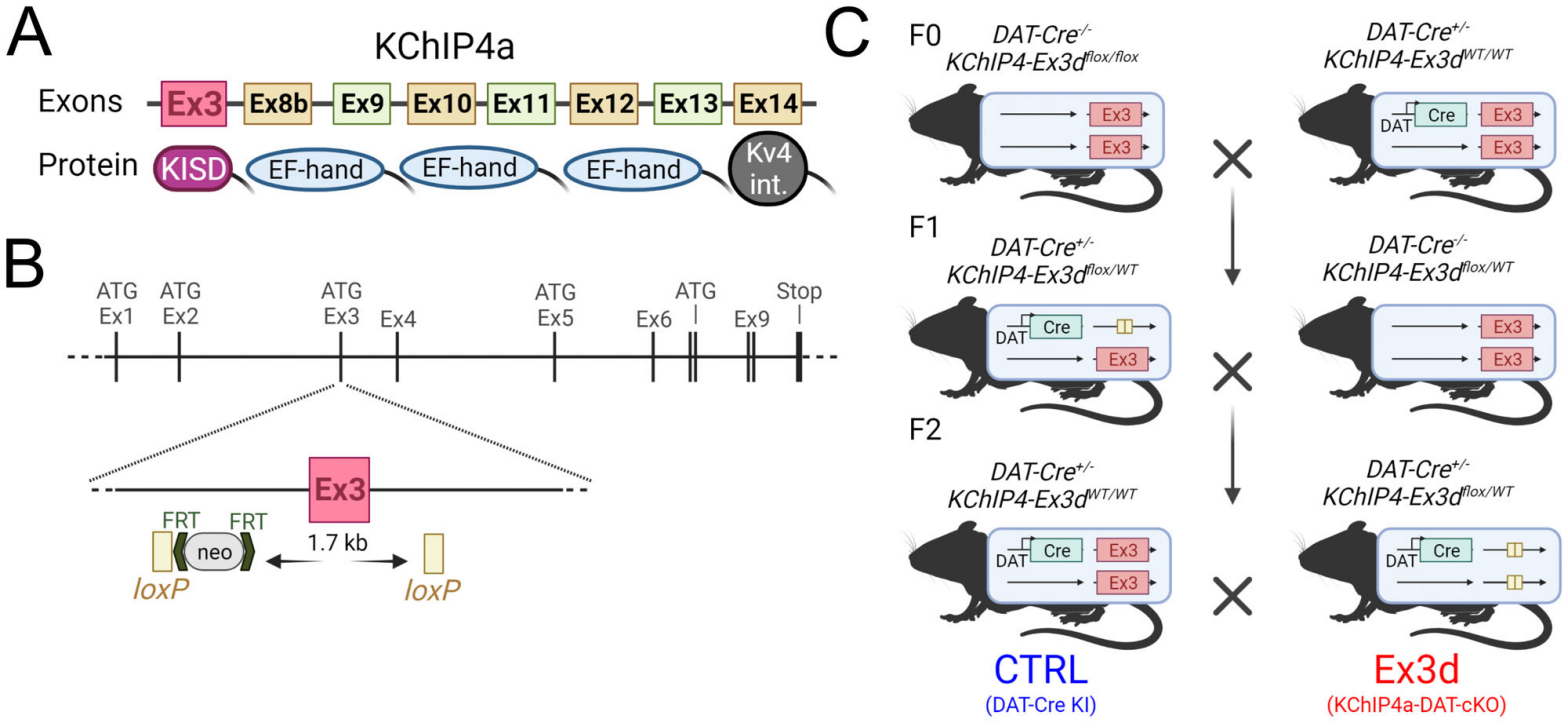
Transgenic strategy for selectively deleting KChIP4a from dopamine neurons. ***A***, Diagram schematic of KChIP4a exons and corresponding proteins. Note that the KChIP4a-defining KID is encoded by Ex3 of the KCNIP4 gene. ***B***, Diagram schematic of the transgenic strategy used for creating the vector where Ex3 was floxed. This vector was later electroporated into mouse embryonic stem cells which were subsequently used to generate mice with a Cre-dependent conditional KO of KCNIP4 Ex3 (see Materials and Methods for details). ***C***, Breeding strategy for generating mice with a deletion of KChip4a only in midbrain dopamine neurons (Ex3d). Conditional KO mice were crossed with DAT-Cre KI mice (F0), generating mice that were double heterozygous or heterozygous only for the Ex3 flox (F1), which were then crossed to produce Ex3d mice and controls that were DAT-Cre KI but did not have floxed KCNIP Ex3 (CTRL).

To selectively knock-out the KChIP4a splice variant only in dopamine neurons, we developed a mouse line where the exon coding for the KID of KCNIP4 (exon 3) was floxed. This exon 3 is unique to the KChIP4a variant (Fig. 1*A,B*; Holmqvist et al., 2002). We crossed these mice with a DAT-Cre line to create mice with a deletion of KChip4a restricted to midbrain dopamine neurons (Ex3d, for “exon 3 deletion”; Fig. 1*C*). For all subsequent procedures, these mice were compared with DAT-Cre^+/−^ littermate controls (CTRL), and mice of both sexes were used for all experiments (Costa et al., 2021).

**Figure 2.**
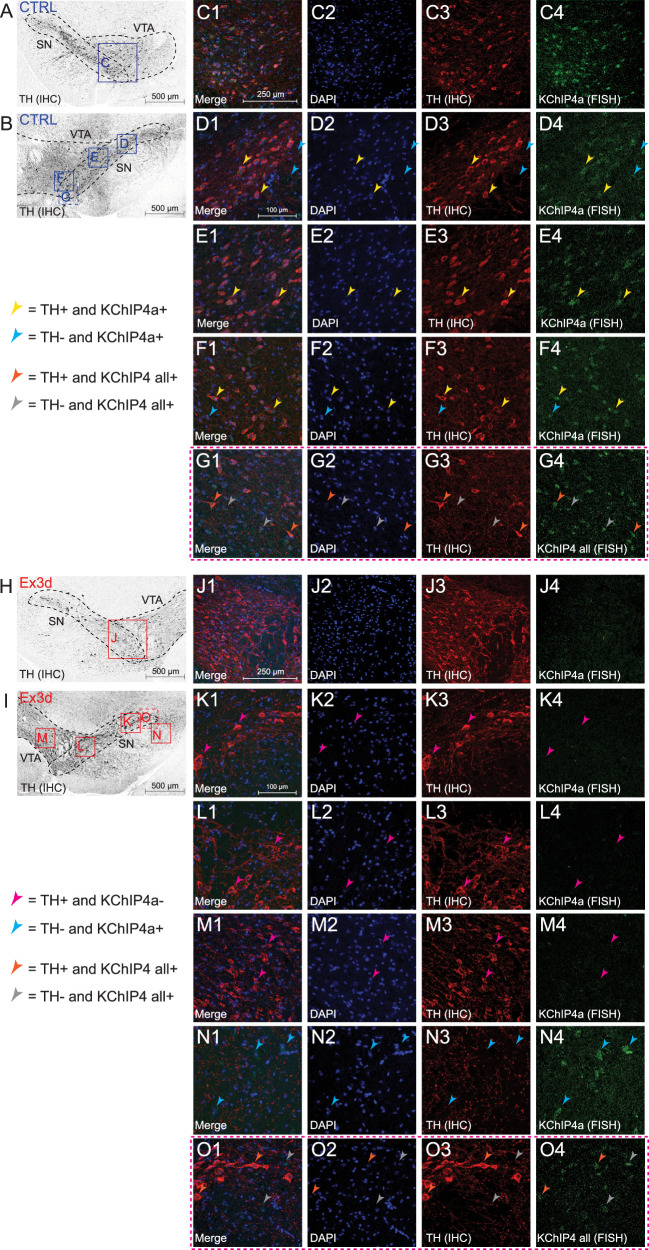
Ex3d mice have a restricted ablation of KChIP4a mRNA expression in dopamine neurons. Combined FISH and IHC assay of CTRL mice showed robust expression of KChIP4a mRNA in midbrain dopamine neurons, while Ex3d mice showed no such expression. ***A***, ***B***, Overview of midbrain slices from a CTRL mouse with a TH where the indicated rectangles represent the ROIs used for panels ***C–G***. ***C–F***, FISH labeling with the KChIP4a-specific probe reveals that CTRL mice show expression of KChIP4a mRNA in TH^+^ and TH^−^ neurons across the midbrain. Yellow arrows indicate select examples of TH^+^ and KChIP4a^+^ neurons; cyan arrows indicate examples of TH^−^ and KChIP4a^+^ neurons. ***G***, Labeling with the general KChIP4 probe, which binds to all KChIP4 isoforms (KChIP4 all), also shows expression of KChIP4 in midbrain TH^+^ and TH^−^ neurons. Orange arrows indicate examples of TH^+^ and KChIP4 all^+^ neurons; gray arrows indicate examples of TH^−^ and KChIP4 all^+^ neurons. ***H***, ***I***, Overview of midbrain slices from an Ex3d mouse where the indicated rectangles represent the ROIs used for panels ***G–K***. ***J–M***, Unlike in the CTRLs, labeling with the KChIP4a-specific probe showed that there was no expression of KChIP4a in TH^+^ neurons across the whole midbrain of Ex3d mice, including the SN and VTA. Magenta arrows indicate select examples of TH^+^ and KChIP4a^−^ neurons. ***N***, However, we did observe robust KChIP4a expression in midbrain TH^−^ neurons, demonstrating that the removal of KChIP4a was cell type specific. Cyan arrows indicate examples of TH^−^ and KChIP4a^+^ neurons. ***O***, Labeling with the general KChIP4 probe showed expression of KChIP4 variants in TH^+^ neurons in the midbrain of Ex3d mice, demonstrating that the removal of KChIP4a was also splice variant specific. *N* = 3 for Ex3d and CTRL.

To validate the effects of our conditional exon-specific knock-out, we combined fluorescent in situ hybridization (FISH) with probes targeting either the KID n-terminal sequence (exon 3, unique to KChIP4a) or the conserved KChIP4 c-terminus (to label all KChIP4 variants), with immunohistochemistry (IHC) for tyrosine hydroxylase (TH) to identify dopamine neurons. We observed clear double-labeling for both exon 3-KChIP4a mRNA and TH in the midbrain of CTRL mice (Fig. 2*A–F*), but no such labeling was seen in Ex3d mice (Fig. 2*H–M*). Labeling of exon 3-KChIP4 mRNA was visible in TH^−^ cells in both CTRL (Fig. 2*F*) and Ex3d mice (Fig. 2*N*), confirming that our mutation ablated KChIP4a mRNA expression only in TH^+^ dopamine neurons. Finally, when using the general, splice variant-independent KChIP4 probe, we observed labeling in TH^+^ cells in both CTRL and Ex3d mice (Fig. 2*G*,*O*), demonstrating that the Ex3d mutation did not eliminate expression of other KChIP4 variants in dopamine neurons.

### Ex3d reduces midbrain KChip4a abundance without affecting overall Kv4 complex composition

**Figure 3.**
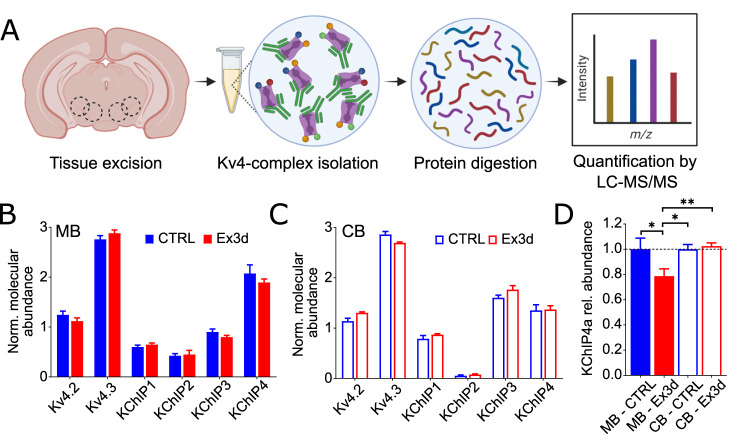
Ex3d mutation reduces KChIP4a protein expression in midbrain dopamine neurons without disrupting overall Kv4 channel subunit composition. ***A***, Diagram schematic of the proteomic analysis of native Kv4 channel complexes. Tissue was excised from ventral midbrain and cerebellum, and Kv4 channel complexes were affinity isolated (antibodies in green, channels and associated proteins in other colors) and analyzed by liquid chromatography-tandem mass spectrometry (LC-MS/MS) following tryptic digest. ***B***, ***C***, Normalized molecular abundance determined for the indicated Kv4 complex constituents from ventral midbrain (MB; ***B***) or from the cerebellum (CB; ***C***) of both CTRL and Ex3d mice (see also Fig. S1). Abundance was normalized to the tetrameric structure of the channel core. Note that Kv4.3 is the predominant pore-forming subunit (3/4 of each tetramer, on average), in both brain regions tested, while KChIP4 is the prevailing auxiliary subunit in MB (2/4 of each tetramer, on average), but not in CB. ***D***, Analysis of the molecular abundance of the KChIP4a protein (relative to CTRL) based on isoform-specific peptides. Note the reduction of KChIP4a abundance in the midbrain of Ex3d mice. Data was analyzed with one-way ANOVA. *N* = 12 experiments for Ex3d and CTRL. Error bars indicate standard error of the mean. *p < 0.05; **p < 0.01.

To assess the effects of the Ex3d mutation on Kv4 channel composition, we performed quantitative high-resolution proteomic analysis of Kv4 channel complexes (Fig. 3) that were affinity isolated from tissue samples excised from the ventral midbrain (region of interest) or cerebellum (control) of both CTRL and Ex3d mice (Bildl et al., 2012; Schwenk et al., 2012; Muller et al., 2016). We found that the average Kv4 channel core in the ventral midbrain is an asymmetric tetramer composed of one Kv4.2 and three Kv4.3 subunits. In the midbrain, this channel core coassembled with an average four KChIP subunits with relative contribution of 0.5 KChIP1, 0.5 KChIP2, 1 KChIP3, and 2 KChIP4 thus revealing KChIP4 as the dominant auxiliary subunit in that region (Fig. 3*A*). In the cerebellum, the relative contribution of KChIP4 was decreased in favor of KChIPs 1 and 3, which means that the high relative abundance of KChIP4 in the midbrain is region specific (Fig. 3*B*). Importantly, there was no significant difference in the relative abundance of all Kv4 complex constituents between CTRL and Ex3d mice, in both midbrain and cerebellum. These findings confirmed that the Ex3d mutation neither disrupts expression of other KChIP4 variants nor does it affect the overall stoichiometry of the native Kv4-KchIP channel complexes.

Next, we determined the relative abundance of KchIP4a in each tissue sample using MS signals of isoform-specific peptides (see Materials and Methods). These analyses revealed a reduction of KChIP4a protein in the midbrain samples from Ex3d mice (Fig. 3*D*). The reduction was not complete (∼25%), likely because nondopamine cells within the midbrain still express KChIP4a in Ex3d mice, which is concordant with a targeted disruption of KChIP4a expression in midbrain dopamine neurons.

### KChIP4a regulates rebound delays and A-type currents in core-projecting dopamine neurons

**Figure 4.**
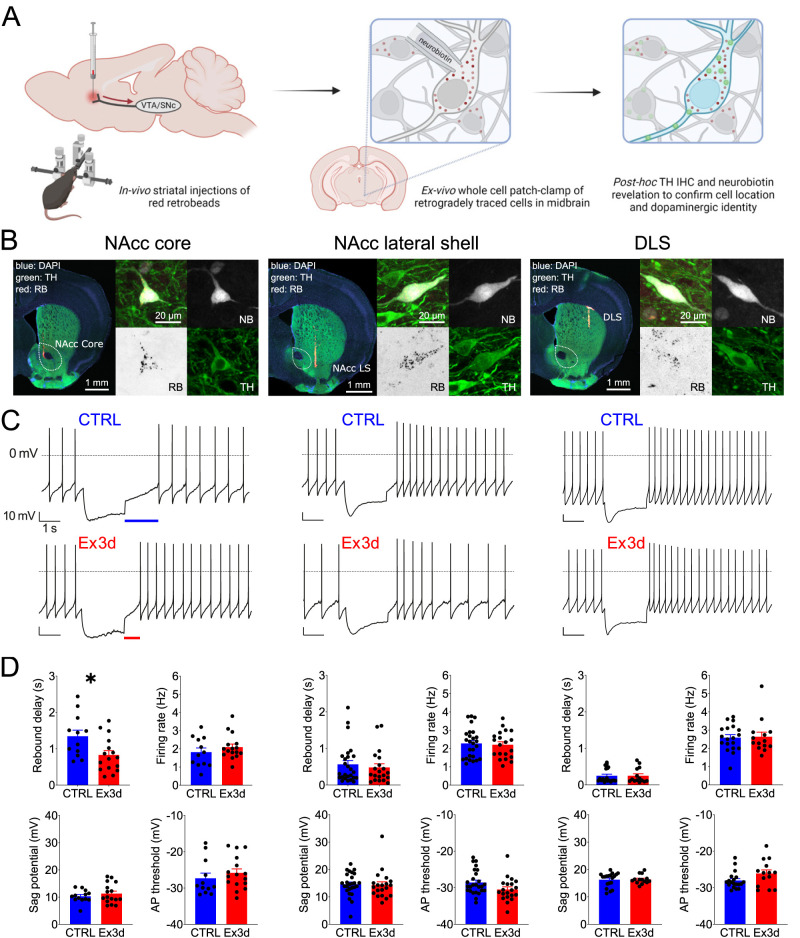
KChIP4a defines the longer rebound delay seen in cNAcc-projecting dopamine neurons. ***A***, Schematic representation of the experimental procedure. Mice, both CTRL and Ex3d, were injected with red retrobeads in different striatal areas, and then retrogradely traced neurons in the midbrain were patched with neurobiotin-filled pipettes. After recordings, brain slices were processed for TH IHC and neurobiotin revelation, to confirm their dopamine phenotype and anatomical location, respectively (see Fig. S2 for retrobead injection sites and recorded neuron locations). ***B***, Representative micrographs of injections in the nucleus accumbens core, lateral shell, and DLS, as well as of recorded, retrogradely labeled, and TH-stained dopamine neurons from each of the projection pathways (see Fig. S2 for the full mapping of injection and recording sites). ***C***, Representative current-clamp recordings of dopamine neurons from both CTRL and Ex3d mice with confirmed projections to the nucleus accumbens core (*n* = 16 cells/*N* = 4 mice for Ex3d and *n* = 12 cells/*N* = 4 mice for CTRL), lateral shell (*n* = 20 cells/*N* = 4 mice for Ex3d and *n* = 27 cells/*N* = 4 mice for CTRL), or DLS (*n* = 14 cells/*N* = 3 mice for Ex3d and *n* = 19 cells/*N* = 3 mice for CTRL), in order (left to right), in response to a −80 mV hyperpolarizing pulse. Note the shorter rebound delay in the nucleus accumbens core-projecting neuron from an Ex3d mouse in relation to CTRL. ***D***, Biophysical properties of projection-identified dopamine neurons from CTRL and Ex3d mice. The only significant difference observed across genotypes and projection targets, using unpaired *t* tests, was a shortening of the rebound delay in DA-cNAcc neurons of Ex3d mice in relation to CTRL. See Figure S3 for more comparisons. AP, action potential. Error bars indicate SEM. *p < 0.05.

To test whether KChIP4a is responsible for the long rebound delays observed in DA-cNAcc neurons (Lammel et al., 2008; Tarfa et al., 2017), we performed in vitro patch-clamp recordings of atypical dopamine neurons from Ex3d and CTRL mice with identified projections to the nucleus accumbens core and compared them to the rebound delays from conventional dopamine neurons that projected to nucleus accumbens lateral shell or DLS. These subtypes were identified by retrograde axonal tracing and post hoc IHC (Fig. 4*A–C*). Similar to what has been reported in previous work (Lammel et al., 2008; Farassat et al., 2019), we found that DA-cNAcc neurons were more concentrated in the medial VTA, DA-lNacc neurons were found more in the lateral VTA and medial SN, and DA-DLS neurons were distributed across the SN (Fig. S2).

We found that the Ex3d mutation selectively reduced the rebound delay in DA-cNAcc neurons by nearly 50% (unpaired *t* test, *p* = 0.016*; Fig. 4*D*), while there was no effect on the rebound delays of the other two projection-defined DA populations (unpaired *t* test, *p* > 0.05; Fig. 4*I*,*J*). This was confirmed with nested analyses that considered individual mice as a factor (Fig. S3)

**Figure 5.**
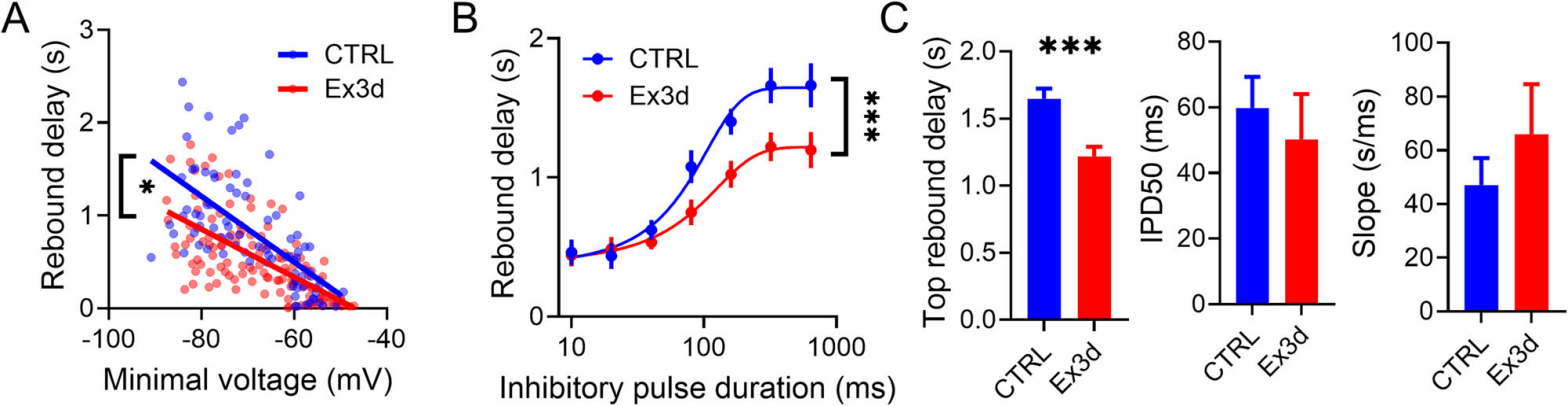
KChIP4a amplifies inhibition in DA-cNAcc neurons in both the amplitude and temporal domains. ***A***, Linear regression plot of minimal membrane voltage obtained during an inhibitory current injection and the resulting rebound delay to firing in DA-cNAcc neurons from CTRL and Ex3d mice. Each data point is one current injection event. Minimal voltage is inversely correlated with rebound delay in both genotypes, meaning that the stronger the hyperpolarization, the longer the following firing pause, but the regression slope Ex3d cells is significantly smaller than for CTRL cells. ***B***, Plot of average rebound delay according to the duration of inhibitory pulses overlayed with a Boltzmann model fit. Rebound delays in Ex3d cells were shorter than those of CTRL cells across nearly all range of pulse durations. ***C***, Boltzmann fit parameters for the data in panel ***B***. Only the maximal rebound delay was significantly reduced in Ex3d cells compared with CTRL cells, indicating that removing KChIP4a reduces the rebound delay across the range of pulse durations without affecting the slope of the duration-rebound relationship. Data from *n* = 15 cells/4 mice for Ex3d and *N* = 12 cells/ 4 mice for CTRL, for both panels 5*A* and 5*B*. See Figure S4 for representative recordings. Error bars indicate SEM. *p < 0.05. ***p < 0.001.

Focusing on Ex3d-induced changes in rebound delay dynamics in the DA-cNAcc neurons, we tested how the depth and duration of inhibition were related to rebound kinetics. For each recorded neuron, we applied negative current steps of 5 or 10 pA up until −80 mV was reached (Fig. S4*A,B*), which led to an average of 7 sweeps per cell (CTRL: 6.8 sweeps, Ex3d: 7.1 sweeps). Plotting the relationship between the minimal voltages in response to the hyperpolarizing current steps and the duration of the rebound delays, we observed a difference in rebound delays between the Ex3D and wildtype DA-cNAcc population (Fig. 5*A*). The correlation slope between minimal voltage and rebound delay was significantly lower in Ex3d neurons (CTRL slope = −3.542*10^−2^ ± 0.461*10^−2^; Ex3d slope = −2.577*10^−2^ ± 0.254*10^−2^; *p* = 0.049*), with similar goodness of fit for both groups (CTRL *r*^2^ = 0.424; Ex3d *r*^2^ = 0.449). This suggests that KChIP4a deletion decreases the rebound delay produced for any given hyperpolarization depth without affecting the basic correlation between these variables.

To test the relationship between the duration of inhibition and subsequent rebound delay in DA-cNAcc neurons, we applied inhibitory pulses of durations between 10 and ∼650 ms and measured the subsequent rebound delay (Fig. 5*B*; Fig. S4*C,D*). The amplitude of the current step was kept constant at the value required to bring the minimum voltage to −80 mV. We found that KChIP4a deletion significantly reduced the rebound delays induced for a range of inhibitory pulse durations consistent with the previous panels (two-way ANOVA; interaction effect, *F*_(6,150)_ = 3.805, *p* = 0.0015**; genotype effect, *F*_(1,25)_ = 5.239, *p* = 0.03*; pulse duration effect, *F*_(2.788,69.69)_ = 5.239, *p* < 0.0001****). Importantly, this effect is seen in response to inhibitory pulses longer than ∼50 ms, suggesting that KChIP4a likely does not affect responses to individual ionotropic inhibitory postsynaptic potentials (IPSPs) even when they would hyperpolarize the membrane potential to −80 mV. Fitting with a Boltzmann function also revealed that this effect was due to an increase in the maximum rebound delay, not in the inhibitory pulse duration needed to reach half-maximum delay, or the slope of the relationship between these variables (Fig. 5*C*). In essence, KChIP4a-containing Kv4 channel complexes effectively amplifies the in vitro rebound delays to sustained inhibition both in the amplitude (>−60 mV) and temporal domains (> 50 ms). This means that, in the presence of KChIP4a, inhibition can be smaller and shorter for similarly effective posthyperpolarization pauses.

**Figure 6.**
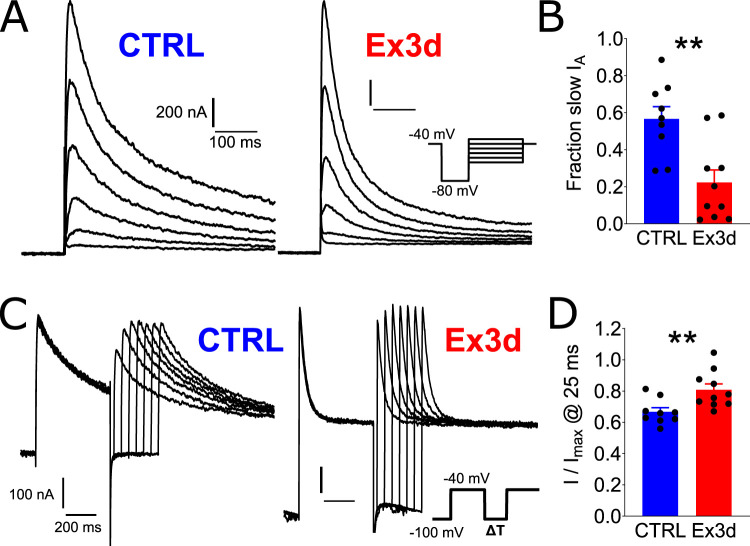
KChIP4a controls A-type current inactivation and recovery in nucleus accumbens core-projecting dopamine neurons. ***A***, Representative recordings of A-type currents (*I*_A_) from CTRL and Ex3d DA-cNAcc neurons, elicited with successively larger depolarization pulses from −80 mV. ***B***, In relation to controls, A-type currents recorded from neurons of Ex3d mice had a significantly lower fraction of slow inactivation, i.e., faster inactivation kinetics, but showed no difference in peak amplitude. ***C***, Recovery from inactivation was tested by applying two 60 mV pulses from −100 mV separated by increasing intervals. ***D***, Compared with CTRL, Ex3d cells showed faster recovery from inactivation. (*n* = 9 cells/*N* = 4 mice for Ex3d and *n* = 11 cells/*N* = 3 mice for CTRL). Error bars indicate SEM. *p < 0.05. **p < 0.01.

Importantly, there was no additional effect on other biophysical properties of DA-cNAcc neurons, like the sag current (Fig. 4*D*, Fig. S3), suggesting that the electrophysiological effects of KChIP4a were not homeostatically compensated, like by Kv4-HCN channel interactions as previously described (MacLean et al., 2005; Amendola et al., 2012). These results confirmed our hypothesis that KChIP4a is a major molecular determinant of extended rebound delays observed in atypical DA-cNAcc neurons. Furthermore, this effect is selective, as other biophysical variables were not affected by the Ex3d mutation. To confirm the biophysical underpinning of the difference in rebound delays, we performed voltage-clamp recordings of A-type currents in DA-cNAcc neurons of Ex3d and CTRL mice. We found that KChIP4a deletion had a dual effect on *I*_A_ kinetics, speeding up both open-state inactivation (*p* = 0.002**; Fig. 6*A*) and recovery from inactivation (two-way ANOVA; interval effect *F*_(3,48)_ = 110.2, *p* < 0.0001***; interaction of interval and genotype effect, *F*_(3,48)_ = 5.321, *p* = 0.003**; *t* test of *I*/*I*_max_ at 25 ms, *p* = 0.0078**; Fig. 6*D*). These results were confirmed with nested analyses (Fig. S5) and are in line with previous reports of KChIP4a’s effect on A-type currents in heterologous systems (Holmqvist et al., 2002; Jerng and Pfaffinger, 2014).

### Computational modeling suggests KChIP4a is a robust amplifier of inhibition

To directly probe whether KChIP4a also controls inhibition in projection-defined atypical DA neurons in vivo in a behaviorally relevant context would require in vivo whole-cell patch-clamp recording from retrogradely traced DA neuron in awake head-fixed mice. As this is currently not yet feasible, we aimed to approach this question by computer simulations of biophysically realistic DA neurons in vivo. Subthreshold input integration in vivo is expected to be different from that observed in ex vivo brain slices because of several factors, including differences in input resistance and the stochastic nature of synaptic inputs (Grace and Bunney, 1983; Otomo et al., 2020). We have recently developed biophysically realistic computational models of atypical dopamine neurons (Knowlton et al., 2021), which include the DA-cNAcc population that we determined to be most affected by KChIP4a deletion. In these models, we can freely explore the effects of any given change in biophysical properties across a wide range of parameters. Therefore, we decided to explore the potential effects of KChIP4a deletion on input processing across different conditions of synaptic connectivity and temporal integration using biophysical models of DA-cNAcc neurons (Knowlton et al., 2021), tailored to reflect the empirically defined membrane properties of these cells with and without KChIP4a deletion. To simulate the effects of binding of different KChIP subunits, we modeled the Kv4.3 current with a Hodgkin–Huxley style (Hodgkin and Huxley, 1952) model with two independent additive inactivation states:

$$I_{\mathrm{Kv}4.3}=g_{\max}p^{3}(\;f_{s}q_{s}+(1-f_{s})q_{f})(E_{K}-V).$$

**Figure 7.**
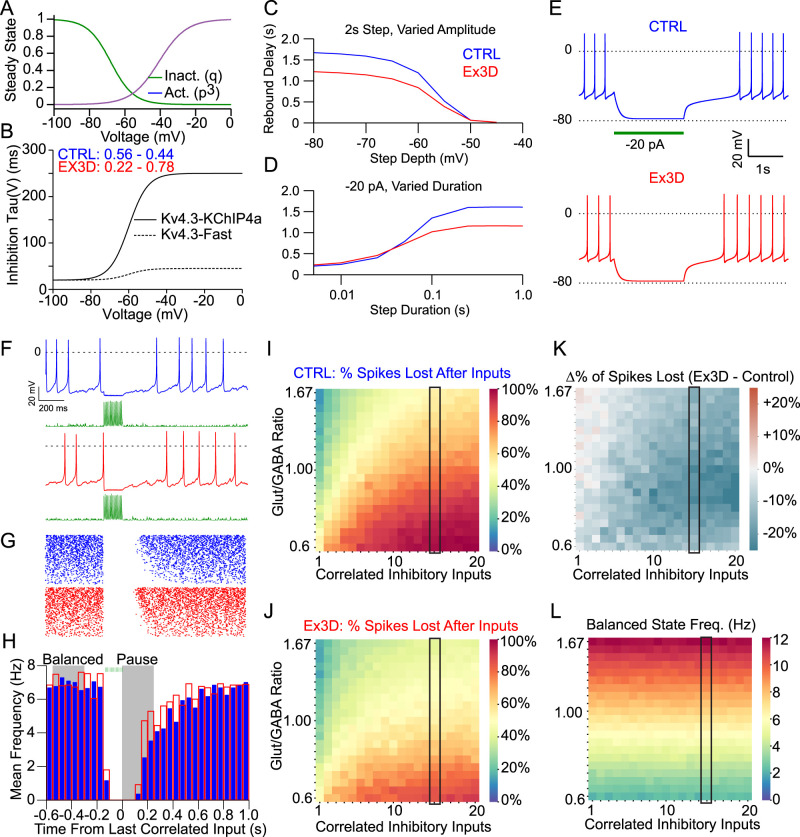
Computational biophysical modeling predicts reduced postinhibitory firing pauses in atypical dopamine neurons after KChIP4a deletion. ***A***, ***B***, Model calibration of Kv4.3 models, including activation and inactivation curves for all Kv4.3 subunits (***A***) and time constant of the inactivation gating variable for KChIP4a (***B***, solid) and fast Kv4.3 subunits (***B***, dashed). ***C***, Replication of the empirical effect shown in Figure 5*A*, which is that KChIP4a removal decreases postinhibitory rebound delays in proportion to the depth of single-step inhibition. All delays follow a 2 s inhibition step to variable depths. ***D***, Replication of the empirical effect shown in Figure 5*B*,*C*, which is that that KChIP4a removal also decreases postinhibitory rebound delays in proportion to the duration of single-step inhibition. All delays follow a −20 pA (−80 mV) step of varied durations. ***E***, Representative traces of the model rebound response from 1 s step to −80 mV for CTRL subunit distribution (blue) and Ex3d subunit distribution (red). ***F***, Similar to ***E*** but in a simulated in vivo condition in a simulated 3.7 Hz balanced state following a correlated train of 100 Hz GABA_A_ IPSC inputs for 250 ms. GABA IPSC inputs are shown in the green traces below. ***G***, Representative raster plots for CTRL (blue) and Ex3d (red) model neurons, with similar GABA input as panel ***G***, but sorted by frequency with slowest at the bottom. The frequency was set by the parameters of the balanced state, and the range is shown in panels ***I–L***. ***H***, Firing frequency histograms after for the same conditions as panels ***F*** and ***G***. Histograms reflect the average of all traces in panel ***G***. Bins are 25 ms intervals averaged over 500 total trials. Density of CTRL (solid blue) overlayed with Ex3d (red box). Gray overlay indicates the periods of balanced state and pause after inhibition. ***I***, ***J***, Percentage of spikes lost relative to baseline after a train of correlated inhibitory inputs for CTRL and Ex3d models, respectively, across several values of glutamate/GABA ratios in the balanced state and of correlated inhibitory input numbers. Black outline indicated the range of glutamate/GABA ratios used for the raster plots in panel ***G***. ***K***, Difference in the percentage of spikes lost between the CTRL and Ex3d models for each combination of balanced state and inhibitory input parameters. Note that the Ex3d model has less spikes lost after inhibition in nearly all possible combinations of balanced state and inhibitory input parameters. ***L***, Mean firing frequency achieved in each balanced state configuration used in panels ***I–L***.

The slow gate (*q*_s_) is associated with the conductance of channels modulated by the KChIP4a subunit, whereas the fast gate (*q*_f_) is associated with the conductance of all other “fast” subunits (Holmqvist et al., 2002; Jerng and Pfaffinger, 2014). The fraction of inactivation that was slow (*f*_s_) was set to 0.56 for control and 0.22 for Ex3d, based on Figure 6*B*. The steady-state values for both inactivation (*q*_f,s_) and activation (*p*; Fig. 7*A*), along with the kinetics of activation, were adapted from Tarfa et al. (2017), and in the absence of subpopulation-specific data, steady-state parameters were held constant across subpopulations. The kinetics of the fast and slow inactivation gates is given in Figure7*A*,*B* and was taken from the voltage-clamp data presented in Figure 6. Representative cells for both CTRL and Ex3d were created using the mean ratios and peak currents from Figure 6*B* for those respective data sets. Consistent with the ex vivo rebound data in Figure 4*C*, the CTRL model cell showed a rebound delay of 1.9 s when released from a 1 s step to −80 mV, while the Ex3d model cell had a rebound delay of 0.97 s under the same conditions (Fig. 7*E*). Consistent with recorded data, the basal firing rate (3.4 Hz in CTRL and 3.3 Hz in Ex3d, on average) was not affected. The model also recapitulated the changes in the relationships between inhibition step depth and duration on rebound delay duration (Fig. 7*C*,*D*), as shown in Figure 5.

Next, we explored how these different Kv4.3 currents affect synaptic integration in a biologically relevant parameter space*.* In behaving animals, hyperpolarization rebound delays are likely to be concurrent with a barrage of both excitatory and inhibitory inputs. Thus, the duration of postinhibitory pauses is not necessarily equivalent to the length of the rebound delay in the low-conductance state ex vivo. Rather, it is shaped by the integration of this rebound with concomitant synaptic input in the high conductance state in vivo. To probe the potential consequences KChIP4a to information processing, we tested how CTRL and Ex3d neurons would respond in a condition that mimicked as closely as possible the synaptic environment in an intact mouse (a “simulated in vivo” model). We did this by applying simultaneous Poisson process trains of excitatory and inhibitory postsynaptic potentials (EPSPs and IPSPs, respectively), to create a balanced state consistent with what has been experimentally observed in dopamine neurons in vivo (Otomo et al., 2020), ex vivo using dynamic clamp (Lobb et al., 2010), and in computational models (Canavier et al., 2016). We then applied phasic inhibitory pulses by introducing temporally correlated GABA_A_ inputs on top of the existing balanced state inputs (Fig. 7*F–L*). Our first immediate observation was that, with the introduction of balanced state inputs and realistic synaptic currents, the examination of individual traces was not sufficient to reveal subtle differences in population responses in most conditions (Fig. 7*F*). Instead, repeated presentations of the stimulus to a single noisy neuron, analogous to trial-based experimental approaches for studying dopamine neuron correlates in reinforcement learning (Schultz et al., 1997), were required to characterize the differences in the response of CTRL and Ex3d cells. That said, Ex3D model neurons clearly showed a larger effectiveness to decrease firing following a train of inhibitory inputs relative to CTRL (Fig. 7*G*,*H*, CTRL in blue and Ex3d in red), consistent with our ex vivo recordings. These differences were found over a wide range of excitation/inhibition ratios (from 0.6 to 1.67; Fig. 7*G*).

Because we do not know the exact synaptic input patterns that are generated during the processing of behaviorally relevant information, we systematically explored how the balance between glutamate and GABA conductances and additional inhibitory GABA IPSC input frequencies could affect neuron firing patterns (Fig. 7*I–L*). This was done by plotting the proportion of spikes that were lost relative to baseline after a train of GABA_A_ inputs at various ratios of the background Poisson barrage of both glutamate and GABA synaptic inputs in CTRL and the Ex3d conditions. This systematic simulation run confirmed that the Ex3d mutation decreased the proportion of spikes lost across nearly all combinations of excitation/inhibition balance and inhibition intensity (Fig. 7*I–K*). Importantly, this was measured relative to baseline firing rates and was consistent across different steady-state firing patterns (Fig. 7*L*). Taken together, our cellular simulation results show that KChIP4a in DA-cNAcc neurons have a selective and robust effect of increasing the efficacy of inhibitory inputs to reduce cell firing across multiple biologically relevant conditions.

### KChIP4a deletion in dopamine neurons enhances learning from negative RPEs

Our electrophysiological and modeling results suggest that KChIP4a acts as a biophysical amplifier of inhibition in DA-cNAcc neurons, which supports our initial hypothesis. The next step was to test whether KChIP4a deletion in dopamine neurons induced a behavioral phenotype that was congruent with this cellular effect. Most prevailing theories of dopamine function posit that cNAcc dopamine signals carry a bidirectional RPE (Schultz et al., 1997; Bayer et al., 2007; Hart et al., 2014) and predict that the shortening of inhibitory firing pauses caused by the Ex3d mutation should also slow down learning from negative RPEs. However, some recent experiments have found that shorter pauses can act as more effective drivers of negative RPE learning than longer pauses (Chang et al., 2016, 2018), and this would predict that the Ex3d mice should show faster learning from negative RPEs. Moreover, another study found that the inhibition of naturally occurring pause duration by selective inhibition of GABA-A receptors, a manipulation that led to similar results on pausing as those derived from our modeling of KChIP4a deletion, enhanced rather than prevented extinction learning (Burwell et al., 2024).

**Figure 8.**
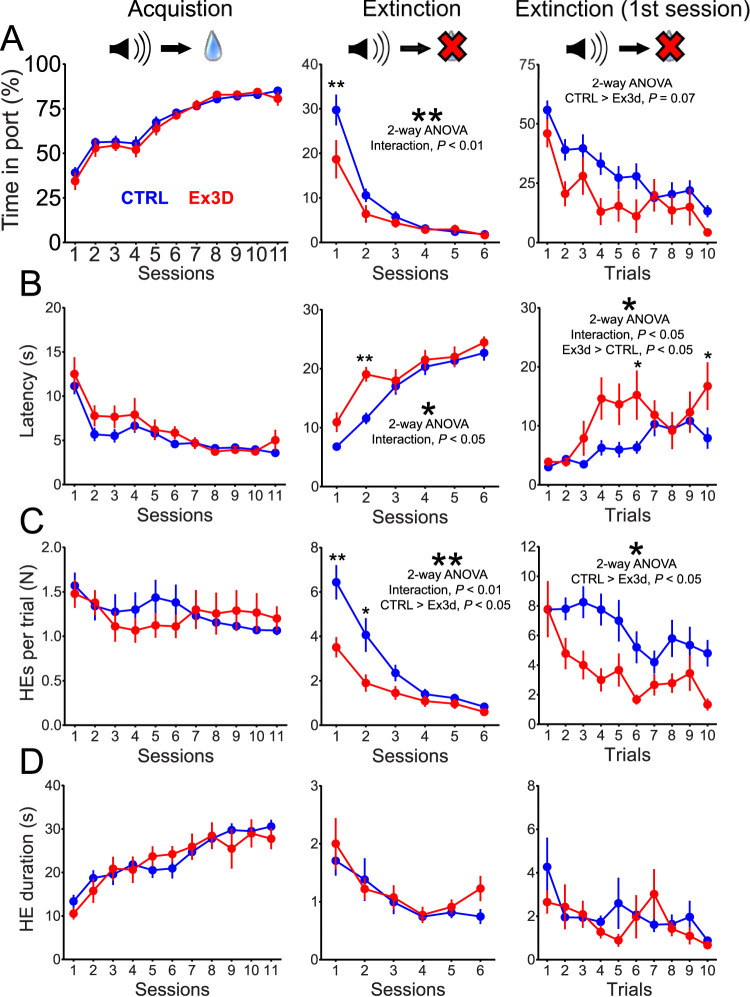
Dopamine neuron-specific KChIP4a deletion improves learning from negative RPEs. ***A***, Time spent in port during CS presentation on a session-by-session basis during acquisition and extinction, as well as on a trial-by-trial basis on the first extinction session. Note the faster response extinction for Ex3d mice in relation to controls, including the significant pairwise difference during the first extinction session, and the strong trend toward faster trial-by-trial extinction in the first extinction session. ***B***, Latency to respond during CS presentation for all phases of the reinforcement learning task. This measure increased faster during extinction in Ex3d mice. Importantly, there was also faster trial-by-trial learning in the first extinction session. ***C***, Number of head entries into the reward port during CS presentation. Note the faster response extinction for Ex3d mice in relation to CTRL, as well as a faster trial-by-trial extinction in the first session of extinction in Ex3d mice. ***D***, Duration of head entries into the reward port during CS presentation. Note the lack of a genotype effect during all phases of acquisition and extinction. Furthermore, for all measured variables, there was no significant difference between genotypes during acquisition or in the first trial of the first session of extinction. *N* = 9 for Ex3d and *N* = 20 for CTRL. Error bars indicate SEM. *p < 0.05; **p < 0.01.

To test between these alternatives, we trained mice on a reinforcement learning task that included both positive and negative RPEs. In this task, mice first learned that an auditory cue signaled the availability of sucrose water reward (acquisition, which depends on positive RPEs), followed by sessions where reward was omitted (extinction, which depends on negative RPEs). Confirming our general expectations, we did not observe a significant difference between genotypes on any behavioral variable during acquisition (Fig. 8*A–D*). However, we found that the Ex3d group had faster extinction learning. Ex3d mice displayed an accelerated reduction in the time they spent in the reward port during cue presentation (interaction of genotype and session progression effect, *F*_(5,135)_ = 3.192, *p* = 0.001**; Fig. 8*A*) and a faster increase in response latency (interaction effect, *F*_(5,135)_ = 2.615, *p* = 0.027; Fig. 8*B*) across extinction sessions. This result is congruent with recent findings that shortening GABA-A-mediated pauses accelerates negative RPE-based learning (Burwell et al., 2024).

To verify whether we were observing a direct effect on learning (which would be compatible with an impact on RPE signaling) or a reduction of responding in the absence of direct reinforcement (more compatible with an impact on motivational processes), we analyzed the trial-by-trial behavior measures in the first extinction session. If the Ex3d mice already displayed a reduced time in port on the first extinction trial (when the mice were first confronted with an unexpected reward omission), then the genotype effect would be more likely attributable to decreased motivation (Berridge, 2012). We found that Ex3d and CTRL mice were statistically indistinguishable in their responses during the first extinction trial (Fig. 8*A–D*). Only in later trials of the first extinction session did genotype differences emerged in response latency (interaction effect, *F*_(9,243(_ = 2.01, *p* = 0.031*; genotype effect, Ex3d > DAT-Cre KI, *F*_(1,27)_ = 7.51, *p* = 0.01*; Fig. 8*B*) and in the number of head entries (genotype effect, CTRL > Ex3d, *F*_(1,27)_ = 5.98, *p* = 0.021*; Fig. 8*C*). We also identified a strong trend for a gradual divergence between genotypes, starting on the second trial, for the time in port metric (genotype effect, CTRL > Ex3d, *F*_(1,27)_ = 3.51, *p* = 0.07; Fig. 8*A*). This confirms that the Ex3d effect developed progressively as the mice learned that the CS no longer predicted US availability, indicating a circumscribed effect on learning from reward omission, i.e., negative RPEs.

**Figure 9.**
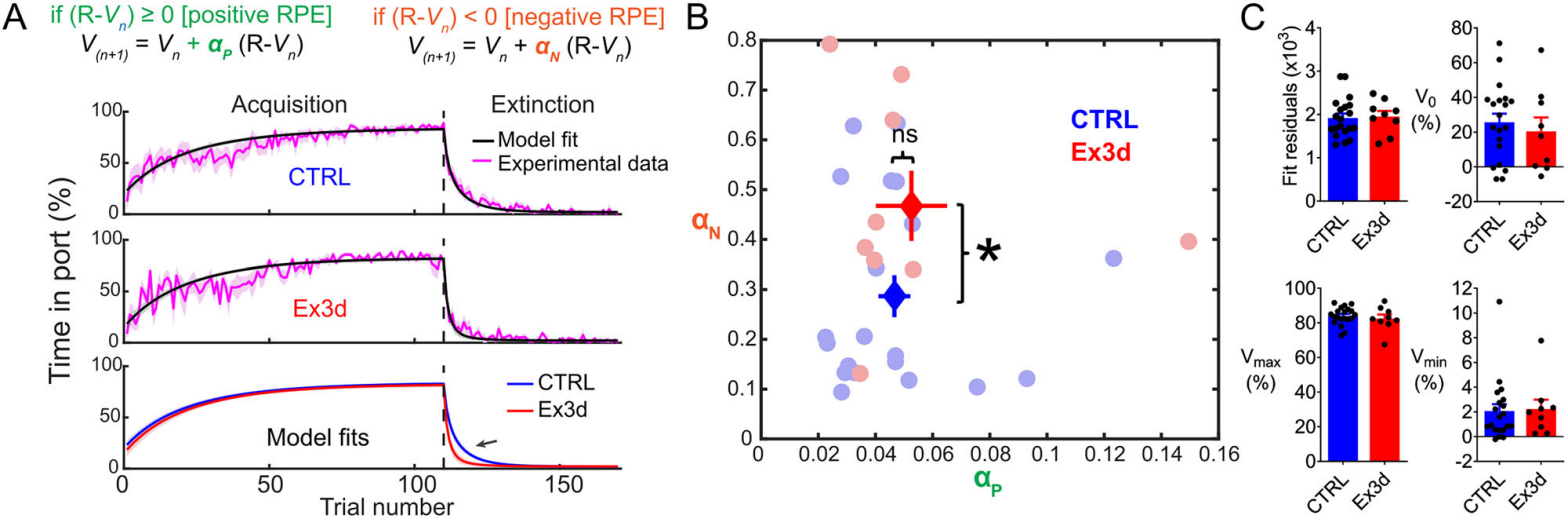
Dopamine neuron-specific KChIP4a deletion improves learning from negative RPEs. ***A***, Fitting of a Rescorla–Wagner model to the trial-by-trial time in port data. Note that the model tracks the behavioral data with relatively good accuracy in both groups and that the model fits overlap almost completely during acquisition, but diverge in extinction, reflecting the faster extinction learning kinetics of the Ex3d mice in relation to controls (black arrow). ***B***, Best fit α_P_ and α_N_ values for Ex3d (red) and CTRL (blue) groups. Circles represent individual values. Crosses indicate the mean ± SEM of each group. Note the selective increase in α_N_ (learning from negative RPEs) in Ex3d in relation to littermate controls. ***C***, *V*_0_, *V*_min_, *V*_max_, and best fit residuals for Ex3d and CTRL groups. No significant difference was observed between the compared groups. *N* = 9 for Ex3d and *N* = 20 for CTRL. Error bars indicate SEM. *p < 0.05.

To formally test that only negative RPE-based learning was enhanced, we fitted the trial-by-trial time in port values for both Ex3d and CTRL groups with a Rescorla–Wagner model which assumed different learning rates for positive (*α*_P_) and negative (*α*_N_) RPE-based learning (Rescorla, 1972; Raczka et al., 2011; Tian and Uchida, 2015). Our model successfully recapitulated the behavioral data, including the different learning kinetics during extinction (Fig. 9*A*). Comparisons of the learning rates for the best fits showed that indeed the Ex3d group had significantly higher *α*_N_ values, i.e., faster learning from negative RPEs (*p* < 0.05), without any change in *α*_P_ (Fig. 9*B*). Importantly, empirically determined model parameters (*V*_0_, *V*_max_, and *V*_min_), as well as goodness of fit, were similar for both genotypes (Fig. 9*C*). These results support the interpretation that the effect of deleting the KChIP4a splice variant in dopamine neurons can be explained by a circumscribed increase in learning from negative RPEs.

**Figure 10.**
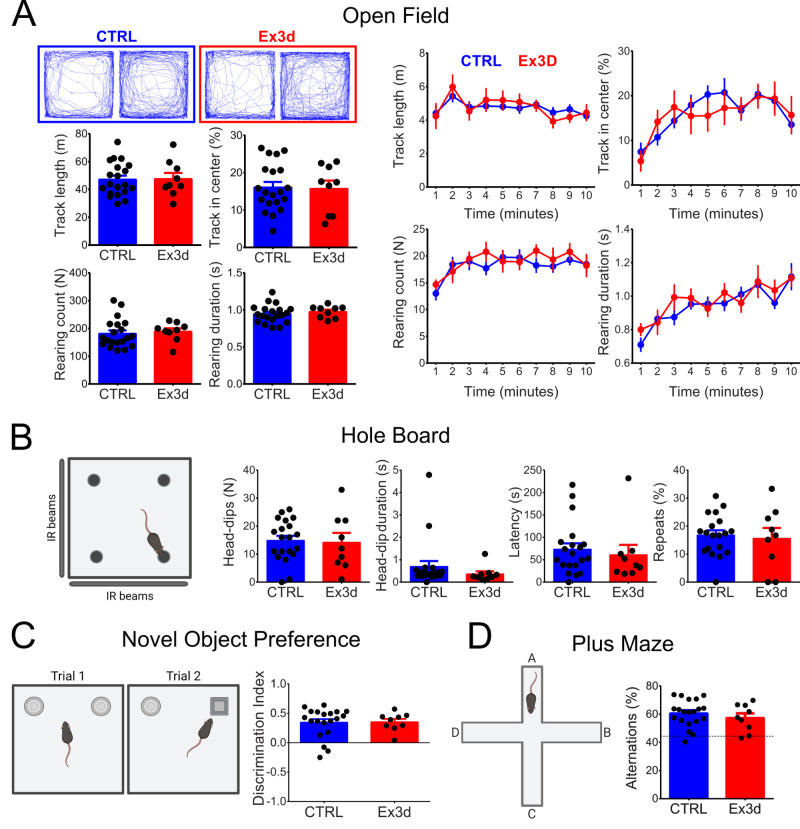
Dopamine neuron-specific KChIP4a deletion does not affect several dopamine-dependent behaviors. ***A***, Session-based results of the open field test, including representative tracks from mice of both genotypes, averages of the total track length, proportion of track length in the center of the open field, number, and duration of rearings during the whole 10 min of the task and averaged across every minute of the 10 min task. ***B***, Hole board test results, including total number of head dips, total summed head dip durations, and latency to first dip and of repeat head dips. ***C***, Diagram representation of the novel object preference test, and the results of the discrimination index (positive values indicate preference for the novel object in Trial 2). ***D***, Plus maze test results; spontaneous alternations were quantified as the proportion of spontaneous alternations relative to the total possible alternations. Note the absence of a significant genotype difference for all variables in all tests (unpaired *t* tests and two-way repeated-measures ANOVA, *p* > 0.05). *N* = 9 for Ex3d and *N* = 20 for CTRL. Error bars indicate SEM.

We also subjected Ex3d mice and CTRL to a battery of dopamine-sensitive tasks to test if KChIP4a deletion in dopamine neurons could have more general effects on behavior. This included open field exploration, hole board exploration, novel object preference, and spontaneous alternation in the plus maze (Fig. 10). There was no difference between the genotypes in any of these tasks, indicating that deleting KChIP4a in dopamine neurons did not impact locomotion, anxiety, spontaneous exploratory behavior, novelty preference and discrimination, short-term memory, or working memory. This series of tests demonstrated that KChIP4a deletion in dopamine neurons does not produce a widespread effect on dopamine-dependent tasks and confirm a selective effect on extinction learning.

## Discussion

In this study, we selectively disrupted the expression of KChIP4a, a splice variant of a Kv4 channel regulatory subunit, in midbrain dopamine neurons. We confirmed this genetic disruption at the level of mRNA expression, Kv4 channel complex composition, and channel biophysics. We found that KChIP4a deletion selectively reduced rebound delays in atypical DA-cNAcc neurons ex vivo. This means that it did not have any other effect on physiological variables within these neurons, nor did it affect any ex vivo properties of conventional dopamine neuron subtypes like DA-lNAcc and DA-DLS cells. This demonstrates that KChIP4a is a molecular determinant of the previously described variability in rebound delays between different projection-defined subpopulations of dopamine neurons (Lammel et al., 2008; Tarfa et al., 2017; Stojanovic et al., 2025). Further analyses revealed that KChIP4a deletion reduced the maximal rebound delays and shifted the time and voltage dependence of rebound delays. These findings establish KChIP4a as a biophysical amplifier of hyperpolarizing inhibition in atypical dopamine neurons. While we have only tested DA-cNAcc neurons, we assume this extends to atypical neurons projecting to medial shell of the nucleus accumbens or prefrontal cortex, as they possess a similar biophysical profile (Lammel et al., 2008).

We used biophysically realistic computational models of atypical DA-cNAcc neurons to simulate the effects of KChIP4a deletion across a wide variety of cellular conditions which are currently unfeasible to test experimentally. The model confirms that shift in inactivation kinetics produced by the removal of KChIP4a is sufficient to reduce rebound delays. We used Poisson-distributed IPSP and EPSP trains to simulate an in vivo-like synaptic state balance and found that KChIP4a deletion reduced rebound delays generated by inhibitory inputs across several combinations of IPSP properties and background excitation/inhibition balance. These computational results support the interpretation that KChIP4a is a selective amplifier of inhibition in atypical dopamine neurons and regulates inhibitory pause durations.

Finally, we found that KChIP4a deletion led to a selective increase in appetitive extinction learning (i.e., learning from negative reward prediction errors), without affecting appetitive acquisition (i.e., learning from positive prediction error) or other dopamine-dependent behavioral variables like working memory and locomotion. Moreover, this effect on extinction learning was circumscribed to changes in the rate with which mice initiated reward-seeking, without affecting disengagement dynamics, suggesting that motivation was also spared (Tecuapetla et al., 2016). Fitting the behavior data to a reinforcement learning model confirmed that this phenotype is compatible with a selective increase in negative RPE learning rates.

Previous studies have linked dopamine firing pauses and dips in accumbal dopamine release to appetitive extinction learning (Pan et al., 2008; Sunsay and Rebec, 2014), and preventing dopamine neuron firing pauses with optogenetic stimulation slows down extinction learning driven by the omission of expected rewards (Steinberg et al., 2013). However, how pause magnitude correlates with extinction learning rate is unclear. Some studies have found that pause duration and firing rate decreases in dopamine neurons, as well as reductions in dopamine signals in the ventral striatum, are positively correlated with probabilistic negative RPE signaling (Bayer et al., 2007; Hart et al., 2014; Tian and Uchida, 2015). These observations support the proposition that dopamine neuron firing and dopamine release in the cNAcc carry a bidirectional RPE, with the duration of pauses being a linear readout of negative RPE magnitude (Dreyer et al., 2010). Our results do not readily fit this framework, as it predicts that removing the KChIP4a-mediated amplification of inhibition in dopamine neurons should slow down, rather than accelerate, extinction learning.

However, some studies using precise optogenetic inhibition of dopamine neurons have suggested that negative RPEs are more effectively signaled by short pauses rather than by long pauses in dopamine neuron firing (Chang et al., 2016, 2018). Even though these studies did not test this effect in simple extinction learning, it is reasonable to believe similar principles may apply to this form of learning. More relevantly, a recent study found that reducing natural phasic inhibition of dopamine neurons via targeted blocking of GABA-A receptors both reduces pause duration in vivo and enhances extinction learning (Burwell et al., 2024). This result is similar to the outcome of our study, where deletion of KChIP4a in dopamine neurons both reduced firing pauses and enhanced extinction learning. Our findings and the results from Burwell et al. (2024) might necessitate a new interpretation of the behavioral significance of pauses in dopamine neurons, as these might not be the main drivers of extinction learning but rather represent a “persistence-despite-failure” signal (Burwell et al., 2024).

Our study has key limitations. First, we have not determined how the observed biophysical changes in DA-cNAcc neurons impact in vivo synaptic integration in dopamine neurons and the subsequent effects on neuronal firing. We also note that even though the fraction of slow A-type current in Ex3d DA-cNAcc neurons is reduced three-fold, strongly supporting our conclusion that KChIP4a is the major determinant of the slowly inactivating Kv4 current in atypical dopamine neurons, it is not entirely abolished, so there may still be other, relatively minor, complementary mechanisms that contribute to this slow inactivation (Jerng and Pfaffinger, 2008). In addition, the Ex3d mutation may also have affected other subtypes of atypical dopamine neurons, such as those that project to the medial shell of the nucleus accumbens (Yang et al., 2018; de Jong et al., 2019), which could have contributed to the observed behavioral phenotype.

Kv4 channels also shape synaptic integration in other cell types. For example, in CA1 pyramidal neurons, Kv4 currents control the amplitude and duration of EPSCs, the backpropagation of dendritic action potentials, and the threshold for LTP induction (Johnston et al., 2003; Kim et al., 2007). Our results add to this literature by demonstrating that KChIP4a modulation of Kv4 channel currents regulates hyperpolarizing inhibition, a feature that may be present in other cell types and used to modulate other behavioral functions. We also highlight that in the process of validating our transgenic model, we discovered that KChIP4 is the most abundant Kv4-binding KChIP subunit in the ventral midbrain, unlike in the cerebellum or other brain regions (Xiong et al., 2004; Jerng and Pfaffinger, 2014). Moreover, our proteomic and FISH results suggest that nondopaminergic neurons in the ventral midbrain also express KChIP4a at relevant levels. One recent study found that in the VTA, glutamate and glutamate-GABA coreleasing neurons, but not GABA-only releasing neurons, also have long rebound delays that are mediated by an A-type current (Miranda-Barrientos et al., 2021). It is therefore possible that these subpopulations of nondopaminergic neurons might have a similar KChIP4a-dependent mechanism for regulating rebound delays, which could be explored in future studies.

The fact that deleting a single splice variant of an ion channel regulatory subunit produced such a congruent and selective phenotype from biophysics to behavior is remarkable. While the function of different neuronal ion channels are thought to overlap to ensure functional robustness (Goaillard and Marder, 2021), our findings suggest that this is not the case for KChIP4a in DA-cNAcc neurons. This could be because the unique properties of KChIP4a are particularly vulnerable to loss of function mutations, which would explain why the structure of KChIP4a is more similar to the earliest known ancestral KChIP than other members of this family (Salvador-Recatalà et al., 2006; Jerng and Pfaffinger, 2014). Atypical neurons, due to their unique ion channel profile (Lammel et al., 2008), may be reliant on KChIP4a as a regulator of inhibitory integration.

Our findings may be relevant for the understanding of disease. Recent studies have found that surviving nigral dopamine neurons of parkinsonian patients and animal models of parkinsonism have increased KCNIP4 expression (Aguila et al., 2021; González-Rodríguez et al., 2021). Furthermore, polymorphisms in the KCNIP4 gene have been linked to different mental illnesses, including substance use disorder (Uhl et al., 2008; Johnson et al., 2011; Han et al., 2013; Gizer et al., 2017), attention-deficit-hyperactivity disorder (Lasky-Su et al., 2008; Neale et al., 2008; Weißflog et al., 2013), depression (Perroud et al., 2012; Ripke et al., 2013), bipolar disorder (Sklar et al., 2008; Xu et al., 2014), autism (Hussman et al., 2011), schizophrenia (Breen et al., 2019; Wang et al., 2023), and personality disorders (Weißflog et al., 2013). Finally, pro-inflammatory stimuli like interleukin-1 alpha can shift the alternative splicing of KCNIP4 to enhance the expression of the KChIP4a isoform (Massone et al., 2011), suggesting a potential connection between KChIP4a and the behavioral consequences of inflammation that should be addressed in future studies.

In summary, we have identified that KChIP4a is a biophysical amplifier of inhibition in atypical dopamine neurons and slows down learning from negative RPEs. Our findings demonstrate the existence of a specialized gene-to-behavior mechanism built within the midbrain dopamine system, shedding light on how the genetically determined biophysical diversity of dopamine neurons supports specific behavioral processes.
